# Gene transfer agents: The ambiguous role of selfless viruses in genetic exchange and bacterial evolution

**DOI:** 10.1111/mmi.15251

**Published:** 2024-03-21

**Authors:** Paul Christopher Michael Fogg

**Affiliations:** ^1^ University of York Biology Department & York Biomedical Research Institute (YBRI) York UK

**Keywords:** bacterial evolution, bacteriophages, DNA packaging, gene transfer agents, horizontal gene transfer

## Abstract

Gene transfer agents (GTAs) are genetic elements derived from ancestral bacteriophages that have become domesticated by the host. GTAs are present in diverse prokaryotic organisms, where they can facilitate horizontal gene transfer under certain conditions. Unlike typical bacteriophages, GTAs do not exhibit any preference for the replication or transfer of the genes encoding them; instead, they exhibit a remarkable capacity to package chromosomal, and sometimes extrachromosomal, DNA into virus‐like capsids and disseminate it to neighboring cells. Because GTAs resemble defective prophages, identification of novel GTAs is not trivial. The detection of candidates relies on the genetic similarity to known GTAs, which has been fruitful in α‐proteobacterial lineages but challenging in more distant bacteria. Here we consider several fundamental questions: What is the true prevalence of GTAs in prokaryote genomes? Given there are high costs for GTA production, what advantage do GTAs provide to the bacterial host to justify their maintenance? How is the bacterial chromosome recognized and processed for inclusion in GTA particles? This article highlights the challenges in comprehensively understanding GTAs' prevalence, function and DNA packaging method. Going forward, broad study of atypical GTAs and use of ecologically relevant conditions are required to uncover their true impact on bacterial chromosome evolution.

## Graphical abstract

Gene transfer agents (GTAs) have been maintained for millions of years as domesticated viruses in the genomes of various prokaryotes. GTAs produced by different species package the entire host chromosome but with species‐specific biases in DNA content. GTAs do not preferentially propagate the genes encoding them and are inherently difficult to identify by bioinformatics, thus their prevalence is unknown. GTAs have been shown to mediate horizontal gene transfer and DNA repair, but the definitive evolutionary benefit of GTA production to the host has not been defined.

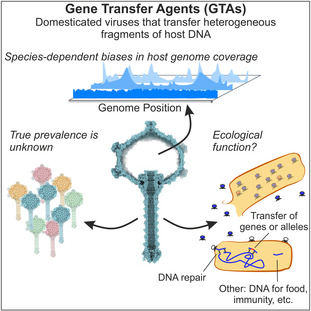

## 1 INTRODUCTION

Bacteria can rapidly exchange or import nucleic acids in response to environmental cues. There are several well‐characterized mechanisms by which bacteria acquire DNA—viral conversion and transduction, competence, and conjugation; however, a chronically understudied mechanism is mediated by gene transfer agents (GTAs). GTAs carry out genetic exchange by packaging short fragments covering the entire bacterial genome, and in some cases extrachromosomal elements, into virus‐like particles or viriforms (Berglund et al., 2009; Gozzi et al., 2022; Hynes et al., 2012; Kuhn & Koonin, 2023; Lang et al., 2012). GTAs are derived from ancient phages and GTA particles are indistinguishable from phages i.e. a DNA filled capsid and associated tail structure. Crucially, the genes that encode GTAs are not preferentially targeted for inclusion into nascent capsids—thus they are essentially *selfless viruses*, that is the majority of DNA within the virus‐derived shell contains non‐self genes and is not used for self‐propagation. The number of genes required to produce a GTA varies from species to species but typically includes a core structural gene cluster (encoding head‐tail proteins) and accessory genes spread across multiple locations in the host genome. The accessory genes encode additional structural proteins, specific regulators, maturation proteins, lysis genes etc (Banks & Le, 2024; Lang et al., 2012). Known GTA particles contain 4–14 kb of DNA; in all cases this is too small to encode all the genes required for GTA production (Banks & Le, 2024; Lang et al., 2012).

GTAs then deliver the packaged DNA to new target cells, where it can be incorporated into the genome or used for DNA repair (Brimacombe et al., 2014, 2015; Gozzi et al., 2022; Tran & Le, 2023). Promiscuous gene transfer could clearly have a major impact on bacterial evolution and/or fitness across different environments, and has been implicated in high frequency gene transfer in the oceans (McDaniel et al., 2010). Indeed there are an estimated 10^31^ phages on Earth, of which a substantial proportion are hypothesized to be GTAs (Hendrix et al., 1999; Kristensen et al., 2010).

## 2 WHAT IS THE TRUE PREVALENCE OF GTAS?

GTAs were originally discovered in the α‐proteobacterium *Rhodobacter capsulatus* B10 (RcGTA) in the 1970s (Marrs, 1974; Wall et al., 1975). RcGTA homologs are present throughout the Rhodobacteraceae where they have co‐evolved with their hosts for hundreds of millions of years, indicative of conserved vertical inheritance (Kogay et al., 2020; Lang & Beatty, 2006; Québatte & Dehio, 2019; Shakya et al., 2017). Furthermore, a recent study used machine learning to massively expand the number of putative GTAs in the α‐proteobacteria based on the characteristic amino acid composition of the head‐tail structural loci (Kogay et al., 2019, 2020; Kogay & Zhaxybayeva, 2022). Strikingly, 57.5% of sequenced genomes (*n* = 1423) contained a *Rhodobacter*‐like GTA structural gene cluster (including the subsequently proven *Caulobacter* GTA (Gozzi et al., 2022)), the majority of which were annotated as prophages (Kogay et al., 2019).

Although this discovery represents a massive increase in the number of potential GTAs, the program was only trained on GTA sequences with sufficient similarity to the canonical *Rhodobacter* GTA and thus more distant GTAs would not be detected. For example, another member of the α‐proteobacteria, *Bartonella*, contains a verified GTA but it is not considered RcGTA‐like (Kuhn & Koonin, 2023; Québatte & Dehio, 2019). Furthermore, beyond the α‐proteobacteria, genetically distinct but functionally analogous GTAs have been discovered experimentally in diverse prokaryotes, including *Brachyspira* sp., *Desulfovibrio* sp. and the archaeon, *Methanococcus voltae* (Bertani, 1999; Eiserling et al., 1999; Lang et al., 2012; Matson et al., 2005; Motro et al., 2009; Rapp & Wall, 1987). These GTAs have received only cursory characterization; typically, involving isolation of the viriform particles, morphology determination by transmission electron microscopy, isolation of packaged DNA and restriction digestion to demonstrate that the content is heterogeneous.

The are several fundamental problems that hinder straightforward bioinformatic identification of GTAs in genome datasets. First, GTAs closely resemble remnant bacteriophages, typically consisting of only the DNA packaging machinery and structural components (Lang et al., 2012). Second, GTAs tend to be encoded by multiple loci, which can be hundreds of kilobases apart and some loci could consist of only one or two genes (Hynes et al., 2016; Québatte et al., 2017; Sherlock & Fogg, 2022a; Stanton et al., 2009; Tomasch et al., 2018). Third, GTAs appear to have evolved multiple times from different ancestral phage, which means that it is not currently possible to use sequence homology, synteny, gene content or structural features to definitively locate novel GTAs. Taken together these factors result in GTAs being misidentified as prophages, or vice versa. Experimental validation of in silico predictions is clearly required to discern between true GTAs and remnant prophages; however, this too is not straightforward. GTAs are usually controlled by interlocking host and/or GTA specific regulators that limit production to a minority of the population (Brimacombe et al., 2013; Farrera‐Calderon et al., 2021; Fogg, 2019; Fogg et al., 2012; Koppenhöfer et al., 2019; Pallegar, Peña‐Castillo, et al., 2020; Québatte et al., 2017; Sherlock & Fogg, 2022a; Westbye et al., 2017), and no clear induction protocols are available for most GTAs.

To truly understand the wider prevalence of GTAs and to deploy sophisticated bioinformatic detection, we require more experimental data about a wider range of GTAs. For example, what fraction of putative GTAs identified by genomics encode functional GTAs? What are the full complements of *Methanococcus* or *Brachyspira* genes responsible for GTA production and regulation? Are these distant GTAs outliers in their respective clades or are homologs prevalent? Are there GTA‐specific characteristics that can be used to differentiate GTAs and phages? Study of diverse GTAs, their structural components and their regulatory pathways will hopefully lead to (A) robust confirmation of predicted GTAs and (B) higher throughput experimental screening via enhanced detection or use of specific induction conditions.

## 3 WHAT IS THE FUNCTION OF GTAS?

Mobile genetic elements that self‐replicate and/or selectively transfer their own genes (phages, plasmids, integrative conjugative elements, transposons, pathogenicity islands etc), actively promote their own survival. Although transfer of host genes may provide a benefit, their mobilization is probably co‐incidental. GTAs, however, do not preferentially transfer the genes encoding them and they require cell lysis for release, which imparts a high cost on GTA producers (Fogg et al., 2012; Gozzi et al., 2022; Hynes et al., 2012; Matson et al., 2005; Tomasch et al., 2018). Therefore, to persist in a population GTAs need to provide a sufficient benefit to outweigh the clear costs of production. It is traditionally thought that the advantages of GTA production are at the population level by facilitating horizontal gene transfer or increased allelic diversity—a kind of primitive sexual reproduction. Are the potential benefits of indiscriminate DNA transfer great enough to explain the persistence of GTAs?

Under laboratory conditions *Rhodobacter*‐like GTAs are primarily produced as the culture enters stationary phase, when the cells are likely to be experiencing stress and nutrients are limited (Québatte & Dehio, 2019; Solioz & Marrs, 1977; Tomasch et al., 2018). In agreement with this, RcGTA and *Dinoroseobacter shibae* GTA (DsGTA) production is controlled by quorum sensing and the stringent response (Fogg, 2019; Koppenhöfer et al., 2019; Leung et al., 2012). Furthermore, chemical inhibition of amino acid synthesis stimulates RcGTA production and a high throughput transposon mutagenesis screen found that RcGTA‐like genes were most beneficial for fitness under nutrient stress—particularly carbon (Kogay et al., 2019; Price et al., 2018; Westbye et al., 2017). Conversely, it should be noted that *Bartonella* GTAs (BaGTAs) are thought to be produced by the fittest sub‐population mediated by ppGpp concentration (Québatte et al., 2017). However, while there is an initial short sharp expression of BaGTAs during exponential growth phase associated with low levels of ppGpp, there is subsequently a more moderate but elongated period of BaGTA production during stationary phase co‐incident with relatively high ppGpp levels. This expression profile is not too dissimilar to RcGTA, although the initial peak of RcGTA production is in late log as cells transition to stationary phase (Solioz et al., 1975). Production of BaGTAs is also closely linked to the expression of various nutrient utilization genes (Québatte et al., 2017).

Could it be that when a population ceases to grow and cell death is inevitable, GTA production allows for the possibility of beneficial allele combinations? Or does sacrifice of the best adapted cells and redistribution of their genes allow the population to thrive? In either case, Redfield and Soucy (2018) argued against the benefit of acquisition of random DNA based on mathematical modeling, in large part due to the cost of producer cell lysis (Redfield & Soucy, 2018). Under favorable conditions and high efficiency of successful gene transfer, the model did allow for a theoretical benefit of GTA DNA recombination. Whether the required level of efficiency is possible under realistic conditions is debateable. Various potential barriers to successful gene transfer have been discussed previously, and below we can consider these in light of recent data.

### 3.1 Loss of GTAs by diffusion

Most known GTA producers are aquatic species and most experimental evidence is based on planktonic growth; under such unconstrained conditions a GTA producer cell would sacrifice itself to release its genome into the milieu where it is unlikely to find a suitable target cell. The host range of GTAs has not been systematically tested, but where data does exist, they can only infect very closely related strains. Recent bioinformatic analyses have pointed to a possible solution i.e. biofilm. In *R. capsulatus*, gene co‐expression and co‐evolution studies have both identified a link between RcGTA genes and biofilm‐associated genes (Kogay & Zhaxybayeva, 2023; Peña‐Castillo et al., 2014). Experimentally, the serine acetyl transferase CysE1 was shown to be involved in regulation of biofilm production and the efficiency of RcGTA receipt (Sherlock & Fogg, 2022b). Intracellular levels of the well‐known biofilm regulator, c‐di‐GMP, are also critical for promotion or inhibition of RcGTA production (Farrera‐Calderon et al., 2021; Pallegar, Canuti, et al., 2020; Pallegar, Peña‐Castillo, et al., 2020; Shimizu et al., 2022; Valentini & Filloux, 2016). Meanwhile, in *Phaeobacter piscinae*, tropodithietic acid (TDA) is a multifunctional secondary metabolite that influences motility, iron uptake and cell morphology. Deletion of the *tdaB* gene abolishes TDA production and also leads to upregulation of the GTA genes and biofilm formation, particularly during initial surface colonization (Lindqvist et al., 2023). These data are consistent with the hypothesis that biofilm could be a natural niche for GTA production where cell density is high and diffusion of GTAs is inhibited, thus increasing the opportunities for successful gene transfer.

### 3.2 Frequency of gene transfer

In vitro experiments have reported that the GTA gene transfer frequencies for specific genes are relatively low (10^−6^–10^−4^ cfu), and in some cases survival in lab media/buffers is poor (Gozzi et al., 2022; Marrs, 1974; Québatte et al., 2017; Solioz & Marrs, 1977). The gene transfer rates observed for GTAs are indeed relatively modest and there also seems to be a plateau effect (Fogg, 2019; Gozzi et al., 2022), however, this can be contrasted with GTA overproduction in the presence of an environmental symbiont (Christie‐Oleza et al., 2015) and phenomenal rates of gene transfer observed in the only large scale in situ study performed to date (McDaniel et al., 2010). Robust GTA stability studies have not been carried out, and it should also be noted that labile viruses under standard lab conditions can often be juxtaposed with high levels of virulence and broad abundance in nature (Bárdy et al., 2023).

### 3.3 Packaging efficiency

A burst size of 100–150 is typical for enterophage T4, however, there is no reason to believe that this is also the case for GTAs (Hadas et al., 1997). Small RNA or ssDNA viruses of similar size to GTAs (~30–40 nm capsid diameter) routinely produce large burst sizes, and even some dsDNA phages are capable of burst sizes orders of magnitude higher than T4 (Pan et al., 2021). Notably, GTAs package significantly less DNA per particle than dsDNA phages with comparable packaging machinery—RcGTA only contains around 4–4.5 kb of DNA. In the absence of DNA replication, GTAs have an absolute burst size limit set by the size of the cell's genome (plus any compatible extrachromosomal DNA) and the capacity of the GTAs e.g. for *R. capsulatus* this equates to a burst size of ~860–968. Furthermore, as GTAs do not appear to require a packaging initiation site (comparable to phage cos, pac etc), they are not likely to be limited to sequential rounds of DNA packaging but can theoretically target multiple different sites on the genome simultaneously thus increasing perceived processivity and consequently burst size. Lack of a typical packaging initiation site could also explain the symmetrical peaks of DNA packaging observed for GTAs as opposed to the unidirectional packaging of phage genomes i.e. once a double strand break occurs the packaging process can proceed in either direction.

### 3.4 Other GTA functions

It is possible that GTAs carry out multiple functions and that the benefit to the host will depend upon the situation the species finds itself in. For example, the recently discovered *Caulobacter crescentus* GTA is capable of effectively repairing DNA damage caused by antibiotics, UV light or restriction enzyme induced double strand breaks; presumably by providing an undamaged copy as a recombination template (Gozzi et al., 2022). Meanwhile, sub‐inhibitory concentrations of the DNA gyrase inhibitor novobiocin led to increased receipt of RcGTAs (Bernelot‐Moens & Beatty, 2022). A role in DNA repair makes sense where DNA transfer can only occur between closely related cells but it is unclear whether this function, or indeed horizontal transfer of genes/alleles, would provide sufficient benefit to outweigh the cost. Furthermore, to observe DNA damage repair, artificial CcGTA overproducer cells were required. Minimal production of CcGTAs occurs under normal growth conditions, which highlights the important problem that most GTA experiments have been carried out under ecologically unrealistic growth conditions or with GTA overproducer mutants (Fogg, 2019; Gozzi et al., 2022; Koppenhöfer et al., 2019). There is clearly a selective pressure for GTAs to arise and to be maintained over long periods of time but establishing the actual benefit GTAs provide to the cell and their true prevalence in nature is core challenge for GTA research.

To determine how active GTAs are in environmentally or medically relevant niches such as soil or animal microbiomes, we require a sustained drive to employ more realistic conditions in the lab, to develop robust in situ studies and to improve environmental detection. More in‐depth study of GTA behavior is crucial: how do GTAs recognize new host cells and how broad is their host range? How large is the GTA burst size, and does this vary from species to species? What are the natural GTA induction conditions? Can we replicate the evolutionary benefits of GTA production under controlled conditions? Are there additional hitherto unknown or unconfirmed benefits conferred by GTAs?

## 4 HOW DO GTAS PACKAGE HETEROGENEOUS DNA?

The packaging of random bacterial DNA by GTAs is fundamentally different to the way bacteriophages and other viruses behave (Rao et al., 2023). Viruses are selfish elements, and their primary aim is to distribute their own genes. Phages first replicate their genome, usually as a multi‐copy concatemer—there is no evidence that GTAs possess any DNA replication genes; instead GTAs appear to directly target the genome of the bacterium that produces them (Lang et al., 2012).

Bacteriophages use a powerful molecular motor complex (terminase) to recognize replicated phage DNA and to drive it into a preformed capsid (Black, 2015). The capsid itself is essentially a passive receptacle and it is the terminase that provides DNA selectivity, enzymatic activity and motive force required to fill the head. RcGTAs (and probably all GTAs), use a headful DNA packaging mechanism i.e. once packaging commences it continues until the capsid is full, at which point the DNA is cleaved and the tail assembly is added (Esterman et al., 2021; Oliveira et al., 2013). Terminases consist of two oligomeric proteins, known as the large (TerL) and small (TerS) subunits. TerL possesses enzymatic activities required for DNA packaging: it has a C‐terminal nuclease domain that cleaves the target DNA to produce a free end available for packaging and an N‐terminal ATPase domain that translocates the DNA into a preformed capsid. TerS is required for regulation of TerL enzymatic activities and recognition of the phage genome i.e. imparting sequence specificity on the packaging machine.

Most characterized phage small terminases have a modular structure: the N‐terminus contains the DNA‐binding domain, the central region contains a coiled‐coil oligomerization domain and the C‐terminus contains the TerL interaction segment. Although, TerS sequences and overall structures vary considerably, the domain organization is conserved (Lokareddy et al., 2022). The RcGTA TerS protein has been shown experimentally to form an oligomer and to directly interact with TerL, however, it appears to lack the N‐terminal DNA‐binding domain (Sherlock et al., 2019). The putative small terminase proteins encoded by known GTAs all lack a detectable DNA binding domain, which holds true even for distantly related species e.g. *Methanococcus* VTA, *Desulfovibrio* Dd1 and *Bartonella* BaGTA. Absence of the specific DNA binding domain is a clear divergence from standard phage terminase structure and could provide an explanation for heterogenous DNA packaging by GTAs. Promiscuous recognition of packaging initiation sites, coupled with headful packaging, could allow GTAs to package the entire host genome with high efficiency and processivity via multiple parallel initiation events.

**FIGURE 1:**
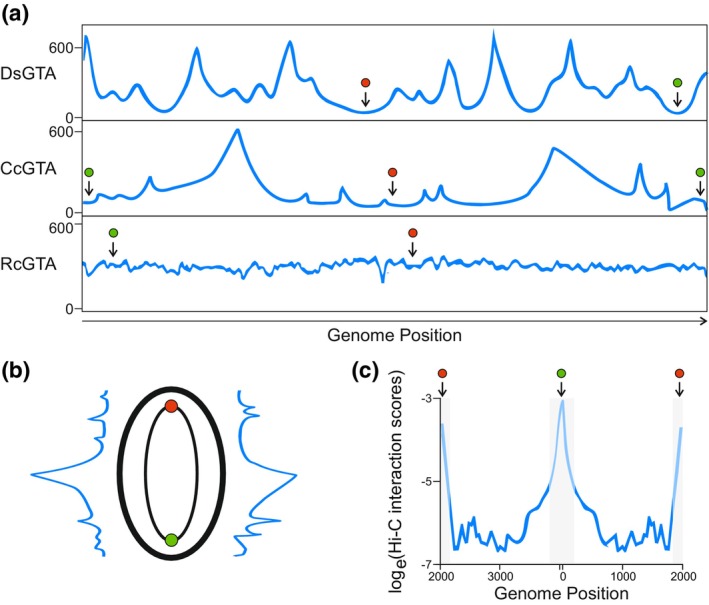
The effect of chromosome organization of gene transfer agents' (GTA) DNA packaging bias. (a) Illustration of the DNA content within GTAs purified from *Dinoroseobacter shibae* (DsGTA), *Caulobacter crescentus* (CcGTA) and *Rhodobacter capsulatus* (RcGTA). Bacterial host genome location is shown on the *x*‐axis and the abundance of sequencing reads mapping to each region on the *y*‐axis. Plots were adapted from Hynes et al. (2012), Tomasch et al. (2018) and Gozzi et al. (2022). (b) Illustration of the *C. crescentus* genome organized longitudinal to the cell, with the CcGTA DNA content relative to the chromosome arms shown on either side. (c) Illustrative plot of interaction scores across the secondary diagonal (inter‐arm interactions) of a *C. crescentus* Hi‐C map. Parts of the plot are grayed to indicate regions with an erroneously high score due to dominant intra‐arm interactions in the circular genome. Adapted from Tran et al. (2017). In all panels, green circles indicate the origin of replication and red circles indicate genome terminal region.

Originally, the DNA content of GTA particles was thought to be essentially random with the entire bacterial genome represented evenly—and this is more or less the case for *R. capsulatus* GTAs (Figure 1a) (Hynes et al., 2012). *Bartonella* GTA DNA has a specific bias radiating from a discrete location in the genome, however this is probably a copy number effect (Québatte et al., 2017). BaGTA production is linked to a run‐off replication (RoR) origin derived from a defective prophage, which amplifies the genomic regions flanking its location leading to their overrepresentation in BaGTA particles. Genes close to the RoR encode various secretion systems (Type III, IV and V) and associated effectors/toxins; transfer of these genes has been suggested to contribute to *Bartonella* host adaptation and virulence (Guy et al., 2013; Québatte & Dehio, 2019).

Despite its similarity to RcGTA, the *D. shibae* GTA (DsGTA) was shown to package DNA with far more specificity; although the entire bacterial genome is still present in DsGTAs, there are also multiple clear peaks of overrepresented regions of the genome indicative of discrete packaging initiation sites (Figure 1a) (Tomasch et al., 2018). *Caulobacter crescentus* GTA (CcGTA) DNA content has an intermediate phenotype with two clear peaks of sequence coverage (Figure 1a) (Gozzi et al., 2022). Could GTA TerS proteins retain sufficient DNA sequence specificity to produce these distinct packaging initiation peaks? It is possible, however, the predicted structures of DsGTA TerS and RcGTA TerS are closely related yet these GTAs produce starkly different DNA packaging profiles (Figure 1a). It is more likely that the RcGTA pattern is due to low level sequence specificity because the peaks are frequent, low prominence and spread across the genome. In contrast, the DsGTA peaks are so prominent and infrequent that they would require strong preference for a few discrete sites. Intriguingly, the *Caulobacter* GTA has no known TerS and deletion of the region immediately upstream of the TerL (the usual location of GTA *terS* genes) did not abolish DNA packaging activity (Tran & Le, 2023).

Tomasch et al. (2018) speculated that the DsGTA DNA packaging biases are the result of unknown chromosome architecture, and supercoiled DNA structures called plectonemes have been described in *C. crescentus* (Le et al., 2013; Tomasch et al., 2018). If *D. shibae*, also has multiple *Caulobacter*‐like plectoneme structures spread throughout the genome then DsGTA targeting of the plectonemes themselves or the intervening plectoneme‐free regions could explain the DNA packaging pattern observed for DsGTA. The *C. crescentus* genome is organized with the origin of replication at one pole, the terminus at the other and the two arms of the chromosome running the length of the cell in parallel (Le et al., 2013). When mapped against the bacterial chromosome, CcGTA DNA has two major coverage peaks roughly in the centre of the two chromosome arms (Figure 1b). The DNA packaging peaks also coincide with the regions of least inter‐arm interactions (Figure 1c). These data seem to argue against the influence of plectonemes, which are distributed frequently across the *C. crescentus* genome and thus one would expect more peaks in the CcGTA DNA (Figure 1). Perhaps the protein occupancy (e.g. SMC, RNApol, ParB etc.) is lower away from the poles and allows easier access to the DNA (Tran et al., 2017). Indeed, it has been suggested that RcGTAs underpackage regions of high expression activity, including the GTA genes themselves, due to conflict with transcription complexes occupying these locations (Hynes et al., 2012). No significant correlation between the biases in DsGTA DNA coverage and transcription was observed, though weak to moderate correlations were noted between troughs of sequence coverage and methylation or GC content (Tomasch et al., 2018). These correlations were not uniform across the genome, which suggests that they are not directly responsible for the packaging bias but may be indicative of underlying factors such as the presence of recently acquired mobile genetic elements.

It would be interesting to see what effect different chromosome organization mutants (SMC complex, HU, *scpA/B* etc) have on CcGTA DNA content i.e. do the coverage peaks grow/shrink, move location or disappear entirely? Moreover, *C. crescentus* is the only species for which data exists on the detailed chromosomal architecture and GTA DNA content (Gozzi et al., 2022; Tran et al., 2017). Production of similar data for *D. shibae* and *R. capsulatus* will be crucial to understand whether chromosome organization plays a role in the respective GTA DNA packaging biases. On balance, the data presented suggests that the absence of a TerS DNA binding domain (or potentially the entire TerS) allows GTAs to package DNA non‐specifically, but it is likely to be chromosome architecture or protein occupancy that leads to DNA content biases.

## 5 ABBREVIATED SUMMARY

GTAs have considerable potential to impact bacterial genetic exchange in the environment and adaptation within ecological niches. Clearly, GTAs are conserved in many bacterial lineages and have also arisen in diverse species by convergent evolution, indicating that they provide a competitive advantage to the host. Study of their evolutionary role is limited and the mechanism by which they provide this advantage is not known.

## AUTHOR CONTRIBUTIONS

**Paul Christopher Michael Fogg:** Conceptualization; funding acquisition; writing – original draft; writing – review and editing.

## ETHICS STATEMENT

There are no ethical implications for this study.

## Data Availability

Data sharing not applicable to this article as no datasets were generated or analysed during the current study.
